# Cytosolic Cadherin 4 promotes angiogenesis and metastasis in papillary thyroid cancer by suppressing the ubiquitination/degradation of β-catenin

**DOI:** 10.1186/s12967-024-05012-1

**Published:** 2024-02-24

**Authors:** Luyao Wu, Jian Xiao, Dandan Yi, Haoran Ding, Ru Wang, Zehua Duan, Zhijian Liu, Xianbiao Shi, Meiping Shen, Jianfeng Sang

**Affiliations:** 1 Division of Thyroid Surgery, Department of General Surgery, Nanjing Drum Tower Hospital, The Affiliated Hospital of Medical School, Nanjing University, 321 Zhongshan Road, Nanjing, 210008 Jiangsu China; 2 Department of General Surgery, The First Affiliated Hospital of Nanjing Medical University, Nanjing, 210029 China

**Keywords:** Papillary thyroid cancer, Cadherin 4, β-catenin, Ubiquitination, Metastasis

## Abstract

**Background:**

Although the long-term prognosis of papillary thyroid cancer (PTC) is favorable, distant metastasis significantly compromises the prognosis and quality of life for patients with PTC. The Cadherin family plays a pivotal role in tumor metastasis; however, the involvement of Cadherin 4 (CDH4) in the metastatic cascade remains elusive.

**Methods:**

The expression and subcellular localization of CDH4 were determined through immunohistochemistry, immunofluorescence, and western blot analyses. The impact of CDH4 on cell migration, invasion, angiogenesis, and metastasis was assessed using transwell assays, tube formation assays, and animal experiments. Immunoprecipitation assay and mass spectrometry were employed to examine protein associations. The influence of CDH4 on the subcellular expression of β-catenin and active β-catenin was investigated via western blotting and immunofluorescence. Protein stability and ubiquitination assay were employed to verify the impact of CDH4 on β-catenin degradation. Rescue experiments were performed to ensure the significance of CDH4 in regulating nuclear β-catenin signaling.

**Results:**

CDH4 was found to be significantly overexpressed in PTC tissues and predominantly localized in the cytoplasm. Furthermore, the overexpression of CDH4 in tumor tissues is associated with lymph node metastasis in PTC patients. Cytosolic CDH4 promoted the migration, invasion, and lung metastasis of PTC cells and stimulated the angiogenesis and tumorigenesis of PTC; however, this effect could be reversed by Tegavivint, an antagonist of β-catenin. Mechanistically, cytosolic CDH4 disrupted the interaction between β-catenin and β-TrCP1, consequently impeding the ubiquitination process of β-catenin and activating the nuclear β-catenin signaling.

**Conclusions:**

CDH4 induces PTC angiogenesis and metastasis via the inhibition of β-TrCP1-dependent ubiquitination of β-Catenin.

**Supplementary Information:**

The online version contains supplementary material available at 10.1186/s12967-024-05012-1.

## Background

Thyroid cancer is a malignant tumor originating from the follicular epithelium or parafollicular epithelium of the thyroid gland [1]. Among these, papillary thyroid cancer (PTC) is the most prevalent, accounting for approximately 70–96% of all cases [2, 3]. In recent years, there has been a rapid increase in the global incidence of thyroid cancer, with females in China ranking fourth among all female malignancies [4]. While most PTC patients have a favorable prognosis, some still experience extensive lymph node metastasis, extrathyroidal invasion, and even distant organ metastasis [5–7]. These factors significantly impact the life quality and overall prognosis of PTC patients [8]. In well-differentiated thyroid cancer, distant metastasis is significantly associated with lower disease-specific survival rates, ranging from approximately 30% to 50%, over a period of five to ten years compared to patients without distant spread [9]. Therefore, exploring the molecular mechanisms underlying PTC metastasis is crucial for identifying molecular targets that can aid in diagnosis and treatment.

Cadherins are the primary adhesion molecules in adherens junctions and play crucial roles in tumor development [10]. Classical type I Cadherins include E-cadherin (Cadherin 1), N-cadherin (Cadherin 2), P-cadherin (Cadherin3), R-cadherin (Cadherin 4, CDH4), and M-cadherin (Cadherin 15) [11]. E-cadherin is recognized for its ability to suppress tumorigenesis and tumor dissemination through intricate mechanisms involving biophysical adhesion processes and mechanotransduction-based intracellular signaling [12]. Conversely, N-cadherin is referred to as a 'mesenchymal cadherin' in carcinomas since it replaces E-cadherin during epithelial-to-mesenchymal transition (EMT) [13]. However, the precise role of other classical type I cadherins, such as CDH4 and P-cadherin, in tumor progression remains elusive [10]. Notably, CDH4 exhibits distinct patterns of cell-specific expression. For instance, in epithelial cell-derived tumors such as breast cancer, gastric cancer, and colorectal cancer, the expression of CDH4 is suppressed due to hypermethylation of its promoter region [14, 15]. Conversely, in osteosarcoma and glioblastoma, there is a significant up-regulation of CDH4 expression [16, 17]. This observation underscores the intricate regulation and context-dependent nature of cadherins in different malignancies. Although we previously demonstrated that CDH4 could promote PTC cell proliferation and invasion while being regulated by long noncoding RNA FER1L4, the role of CDH4 in PTC progression and metastasis is still unclear [18].

β-catenin, a versatile and evolutionarily conserved molecule, plays pivotal roles as an essential structural component of cadherin-based adherens junctions and the central nuclear mediator of canonical Wnt signaling [19]. Newly synthesized β-catenin forms a complex with E-cadherin at adherens junctions, where it regulates the actin cytoskeleton dynamics [19]. In the cytoplasm, β-catenin undergoes degradation mediated by a multiprotein "destruction complex" comprising tumor suppressors Axin and adenomatous polyposis coli (APC), Ser/Thr kinases GSK-3 and CK1, the E3-ubiquitin ligase β-transducin repeat-containing protein (β-TrCP), and protein phosphatase 2A (PP2A) [20]. Upon stimulation by Wnt, the destruction complex of cytoplasmic β-catenin is inhibited, thereby facilitating the accumulation and translocation of β-catenin into the nucleus. Subsequently, within the nucleus, β-catenin orchestrates the transcriptional regulation of Wnt target genes [21]. Augmented nuclear localization of β-catenin exerts influence on diverse cellular processes, such as disruption of intercellular adhesion and enhancement of cell migration potential [21]. Although CDH4 belongs to the classical type I cadherin family and shares a similar structure with E-cadherin, the interaction between CDH4 and β-catenin remains undisclosed.

​In this study, CDH4 was found to be upregulated and exhibit cytoplasmic localization in PTC tissue. CDH4 promoted PTC cell migration and invasion, angiogenesis, and metastasis. Mechanistically, cytosolic CDH4 interacted with β-catenin and impeded β-TrCP1-mediated ubiquitination and degradation of β-catenin, thereby facilitating the nuclear translocation of β-catenin and enhancing the transcription of downstream genes. Thus, our findings unveil a novel mechanism implicated in tumor metastasis wherein the CDH4/β-catenin complex assumes an indispensable role, underscoring the potential of targeting CDH4 as a viable approach to modulate β-catenin in cancer therapy.

## Materials and methods

### Tissue samples and cell lines

Tumor and adjacent normal tissues were obtained from 17 patients with PTC who underwent thyroidectomy at the Nanjing Drum Tower Hospital. Moreover, a PTC tissue array (n = 29) was purchased and processed from Servicebio (Cat. No. TC-1503, Wuhan, China). Before surgery, all patients had not received any treatment. The collected tissue samples were promptly frozen in liquid nitrogen and stored at − 80 °C until further analysis. This study was ethically approved by the Ethics Committee of Nanjing Drum Tower Hospital, and written informed consent was obtained from all participants before specimen collection.

Four PTC cell lines (TPC-1, BCPAP, KTC-1, and IHH-4) and a normal thyroid follicular epithelium cell line (Nthyori3-1) were purchased from the Cell Bank of Type Culture Collection of the Chinese Academy of Sciences (Shanghai, China). KTC-1, BCPAP, and Nthy-ori3-1 cells were cultured in RPMI1640 medium (Cat. No. 11875093, Gibco, Carlsbad, CA, USA), while TPC-1 cell and human umbilical vein endothelial cells (HUVECs) were cultured in DMEM with high glucose (Cat. No. 11965092, Gibco, Carlsbad, CA, USA). All cell lines were incubated in a humidified atmosphere at 37 °C containing 5% CO2.

### Plasmids, siRNAs, and cell transfection

siRNA oligonucleotides targeting CDH4, along with negative control siRNA, were procured from GenePharma (Shanghai, China). Lentiviral constructs that express the full-length CDH4 or shRNA sequences that targeted CDH4 were generated by cloning the corresponding sequences into the pCDH-CMV-MCS-EF1-Puro vector. The HA-Ub plasmid (pCMV-HA-Ub), His-CDH4 plasmid (pCMV-6 × His-CDH4), and Flag-β-catenin plasmid (pCDEF-FLAG-β-catenin) were obtained from Miaoling (Wuhan, China). Transfection techniques adhered to previously reported methods and manufacturer's instructions [18]. The specific sequences of the siRNA or shRNA mentioned above can be found in Additional file 1: Table S1.

### RNA extraction, reverse transcriptional PCR, and quantitative real-time PCR

Total RNA was extracted from cultured cell lines using TRIzol reagent (Cat. No. 15596026CN, Invitrogen, MA, USA). Subsequently, cDNA synthesis was performed utilizing the PrimeScript RT reagent kit (Cat. No. RR047A, Takara, Kyoto, Japan). For quantitative real-time PCR (qRT-PCR) analysis, AceQ qPCR SYBR Green Master Mix (Cat. No. Q131-02, Vazyme, Nanjing, China) was employed. The obtained results were evaluated employing the 2^−ΔΔCT^ method and relative expression levels were normalized to the internal control GAPDH. Primers used in qRT-PCR were as follows: MYC proto-oncogene, bHLH transcription factor (c-Myc): Forward 5′-AAAGGCCCCCAAGGTAGTTA-3′, Reverse 5′-GCACAAGAGTTCCGTAGCTG-3′; matrix metallopeptidase 7 (MMP7): Forward 5ʹ-CATGATTGGCTTTGCGCGAG-3ʹ, Reverse 5ʹ-AGACTGCTACCATCCGTCCA-3ʹ; vascular endothelial growth factor C (VEGFC): Forward 5′-GGCTGGCAACATAACAGAGAA-3′, Reverse 5′-CCCCACATCTATACACACCTCC-3′; glyceraldehyde-3-phosphate dehydrogenase (GAPDH): Forward 5′-TGCACCACCAACTGCTTAGC-3′, Reverse 5′-GGCATGGACTGTGGTCATGAG-3′.

### Immunofluorescence (IF)

Immunofluorescence was performed by a previously established protocol [22]. Briefly, cells were fixed and then incubated overnight at 4 °C with primary antibodies. Immunoreactivity was visualized using FITC- or Cy3-labeled Goat Anti-Rabbit/Mouse IgG secondary antibodies. Subsequently, DAPI was used for nuclear staining, and images were captured under an immunofluorescence microscope (THUNDER DMi8, LEICA, German). Using ImageJ software, the fluorescence of CDH4 or active β-catenin was converted into pixels, and IntDen (area × mean grey value), an indirect assessment of protein expression, was calculated. Colocalization between the signals from CDH4 and β-catenin was also quantified using the Manders correlation coefficients. The Coloc 2 program from ImageJ/Fiji was used for quantification of the colocalization.

### Immunohistochemistry (IHC) analysis

Human PTC tissues and subcutaneous tumor tissues from nude mice were fixed using 4% paraformaldehyde. Paraffin sections were prepared, followed by antigen retrieval with EDTA solution after dewaxing treatment. The tissues were incubated overnight at 4 °C with primary antibodies. Images were captured using an upright microscope (Olympus). The evaluation criteria for staining intensity are as follows: 0 point for no staining, 1 point for yellow, 2 points for brown and yellow, and 3 points for yellowish brown. The scoring system for the proportion of positively stained tumor cells is as follows: 0 (no positive tumor cells), 1 (< 10%), 2 (10–50%), and 3 (> 50%). The IHC score was calculated as staining intensity × percentage of positive tumor cells, resulting in scores as 0, 1, 2, 3, 4, 6, and 9 [23]. A score ranging from 0 to 3 was designated as indicative of low expression, while a range of 4 to 6 denoted moderate expression. A score of 9 indicated high expression.

### Protein extraction and immunoprecipitation (IP)

Proteins from whole cell lysates (WCL), cytomembrane (CM), cytoplasm (CP), and cell nucleus (CN) were extracted using the NP-40 lysis buffer (Cat. No. P0013F, Beyotime, Shanghai, China), Membrane and Cytosol Protein Extraction Kit (Cat. No. P0033, Beyotime, Shanghai, China), or Nuclear and Cytoplasmic Protein Extraction Kit (Cat. No. P0028, Beyotime, Shanghai, China), respectively.

For immunoprecipitation, 500–1000 μg of cell lysate samples were mixed with specified antibodies and incubated at 4 °C overnight. Next, the sample lysate was mixed with protein A/G magnetic beads (Cat. No. PB101-01, Vazyme, Nanjing, China) and incubated at room temperature for 30 min. Finally, 1 × SDS-PAGE loading buffer was added to re-suspend the magnetic beads and heated at 95 °C for 5 min. The supernatant was collected for subsequent SDS-PAGE detection.

### Mass spectrometry analysis

After performing IP and fractionation on SDS-PAGE, the proteins were subjected to silver staining (Cat. No. P0017S, Beyotime, Shanghai, China). Subsequently, target strips were carefully excised and subjected to mass spectrometry analysis using a Tandem mass spectrometer (Q-Exactive HF X, Thermo Fisher Scientific, San Jose, CA) at BGI (Shenzhen, China).

### Antibody and western blot (WB) analysis

In brief, the protein sample (20 μg) firstly underwent electrophoresis in a tris–glycine electrophoresis system, followed by transfer of the target bands onto polyvinylidene fluoride membranes (PVDF), and visualization was achieved using a chemiluminescent detection system. The detailed information on antibodies is summarized in Additional file 1: Table S2.

### Protein stability assay

To determine the half-life of β-catenin, cells were treated with a protein synthesis inhibitor cycloheximide (Cat. No. 239765, Sigma-Aldrich, Darmstadt, Germany) at a concentration of 100 µM for the specified durations. Protein levels were assessed by Western blot.

### Ubiquitination assay

For in vitro ubiquitination assays, the HA-Ub plasmid was transfected into PTC cells for 48 h and treated with MG132 (20 μM) for 6 h. The cells were harvested and lysed with NP-40 lysis buffer (50 mM Tris (pH 7.4), 150 mM NaCl, 1% NP-40) containing protease inhibitors. Then, 500–1000 μg of cell lysate samples were mixed with 5 μg anti-β-catenin antibody and placed on a vertical rotating homogenizer at 4 °C overnight to form an immune complex. On the second day, magnetic beads were added to the immune complex and incubated at room temperature for 30 min. Then washing buffer (1 × PBS, 0.5% Tween 20, pH 7.4) was added to re-suspend the magnetic beads. Finally, 1 × SDS-PAGE loading buffer was added and heated at 95 °C for 5 min. The supernatant was collected for subsequent SDS-PAGE detection. The ubiquitination level of β-catenin was detected by Western blot using anti-HA antibody.

### HUVECs tube formation assay

HUVECs (1 × 10^4^ cells per well) suspended in the conditional medium were added to a 96-well plate pre-coated with 50 μL Matrigel (Cat. No. 356230, Corning, NY, USA). Following a 4-h incubation at 37 °C, the establishment of an interconnected network was observed under microscopic examination. The quantification of total branching tube lengths per field was performed using the Angiogenesis Analyzer plugin integrated into the Image J software platform.

### Animal experiments

In this study, four-week-old female BALB/c nude mice were obtained from the Experimental Animal Center of Nanjing Medical University and housed in specific pathogen-free barrier facilities. To establish the lung metastatic model, 2 × 10^6^ TPC-1 cells were directly injected into the tail vein of nude mice. Approximately six weeks later, euthanasia was performed on the mice, and their lungs were subjected to hematoxylin and eosin (H&E) staining. For the tumorigenicity studies, TPC-1 cells (2 × 10^6^ cells) were subcutaneously inoculated into the side armpit of nude mice. Tumor growth was monitored weekly using a caliper and calculated using the formula: volume = (length × width^2^)/2. After eight weeks post-injection, euthanasia was conducted on the mice. In order to evaluate Tegavivint's effects (Cat. No. S0733, Selleck, Shanghai, China), a group of mice received intraperitoneal injections twice weekly for three consecutive weeks per cycle with a dosage of 20 mg/kg (n = 5), repeated over two cycles lasting 28 days each [24]. All animal experiments were approved by the Committee on the Ethics of Animal Experiments of the Nanjing Medical University.

### TOP-flash/FOP-flash luciferase reporter assay

Cells were co-transfected with TOP flash or FOP flash expression plasmid (Beyotime, Shanghai, China) using Lipofectamine 3000. Luciferase activity was assessed utilizing the Dual Luciferase Reporter Assay (Cat. No. E2920, Promega). Firefly luciferase activity was normalized to Renilla luciferase activity, and the results were presented as the normalized TOP/FOP ratio.

### Cell migration and invasion assays

Cell migration and invasion were assessed using transwell chambers with 8 μm pores (Corning, NY, USA). For the cell migration assays, treated cells (2 × 10^4^ cells/well) suspended in 200 μL serum‐free medium were added to the upper chambers. The lower chambers were supplemented with 500 μL complete culture medium as a chemoattractant. In terms of invasion assays, cells were seeded on Matrigel-coated upper chambers. The detailed protocol has been described previously [18].

### Statistical analysis

The experimental data were presented as mean ± standard deviation (SD) and subjected to statistical analysis using GraphPad Prism 6 (GraphPad Software, San Diego, CA, USA). Student's t-test (two-tailed) was used to compare two groups, while ANOVA was employed for comparing multiple groups. Clinicopathological results were compared utilizing Pearson χ2 tests. A significance level of *P* < 0.05 was considered statistically significant.

## Results

### CDH4 is upregulated in PTC and predominantly localized in the cytoplasm

**Fig. 1:**
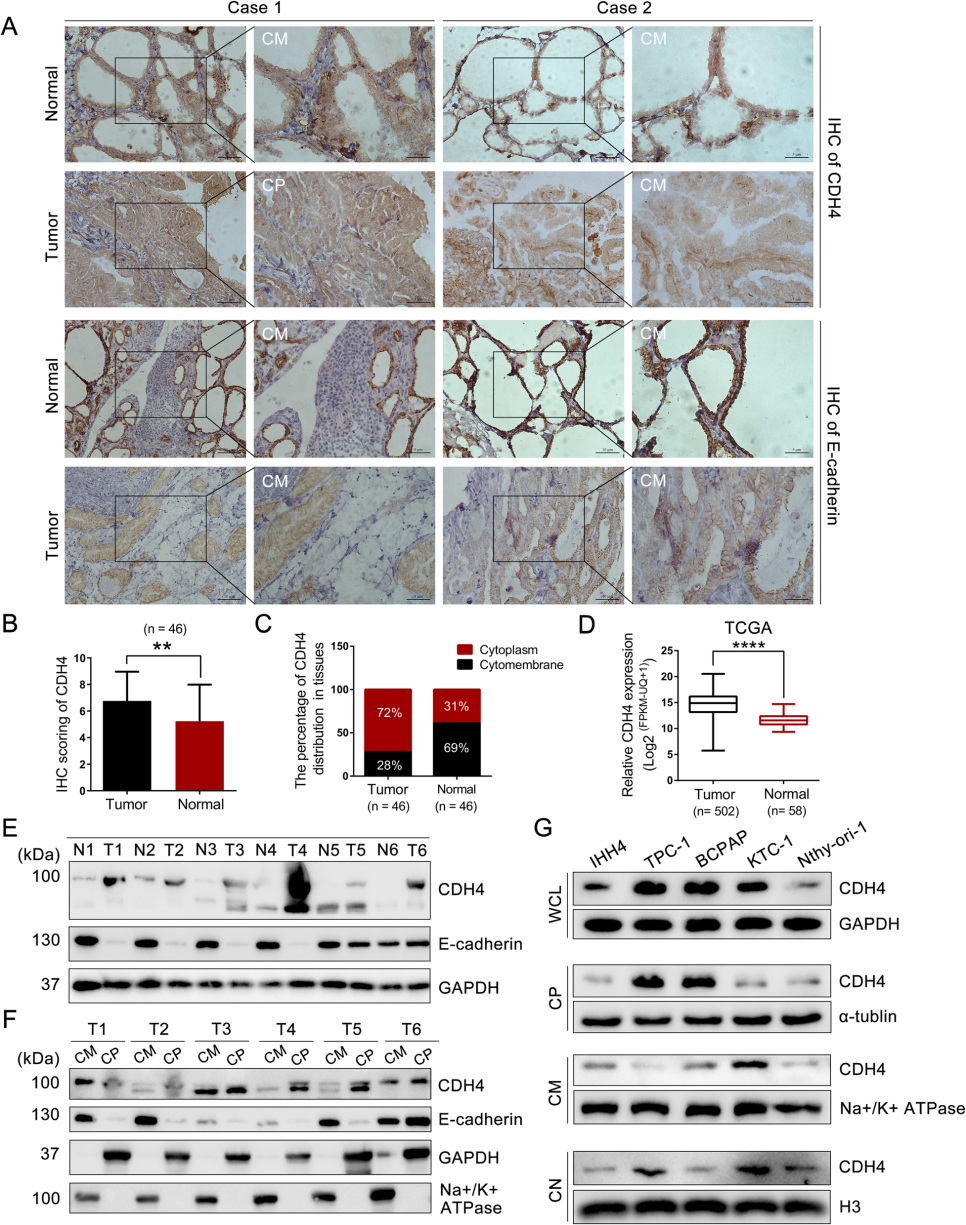
CDH4 is upregulated in PTC and predominantly localized in the cytoplasm. **A** Representative IHC images of CDH4 and E-cadherin in PTC tissues and adjacent normal tissues. *CM* cytomembrane, *CP* cytoplasm. **B** The quantification of CDH4 expression in 46 pairs of PTC tissues. **C** The percentage of CDH4 localized in the cytomembrane or cytoplasm in tumor tissues and adjacent normal tissues. **D** The expression of CDH4 in 502 PTC tissues and 58 normal thyroid tissues from the TCGA database. **E** WB analysis of CDH4 and E-cadherin expression in paired PTC tissues and normal tissues. **F** Detection of the expression level of CDH4 in the cytomembrane or cytoplasm in six PTC tissues. GAPDH and Na + /K + ATPase were used as the internal control for cytosolic proteins or cytomembrane proteins, respectively. **G** The expression patterns of CDH4 in PTC cell lines and Nthy-ori-1 cells. **P < 0.01; ****P < 0.0001

Firstly, in the 17 pairs of PTC tissues collected at our hospital, a higher expression of CDH4 was observed in tumor tissues through IHC analysis. Intriguingly, it was discovered that CDH4 predominantly exhibited cytoplasmic distribution within tumor tissues while primarily localized to the cytomembrane in adjacent normal tissues (Fig. 1A). Correspondingly, E-cadherin demonstrated predominant membranal localization in both tumor and normal tissues; however, its expression level was significantly diminished in tumor tissues compared to normal tissues (Fig. 1A). To validate this result, IHC analysis was conducted on a PTC tissue array (n = 29) (Additional file 1: Fig. S1A). Based on these two IHC experiments, a notable increase in CDH4 expression and its cytoplasmic localization was observed in tumor tissues when compared to adjacent normal tissues. (Fig. 1B, C). Using the RNA-seq data from the Cancer Genome Atlas Program (TCGA), we also found a higher expression level of CDH4 in tumor tissues than in normal tissues (Fig. 1D). Additionally, Western blot analysis conducted on six pairs of PTC tissues also revealed an upregulation of CDH4 and a downregulation of E-cadherin in tumor tissues (Fig. 1E). Next, we extracted proteins from the cytomembrane or cytoplasm of PTC tissues to further analyze the subcellular distribution of CDH4. It showed that CDH4 was predominantly localized within the cytoplasmic compartment (Fig. 1F). Importantly, in comparison to the immortalized thyroid follicular epithelium cell line Nthy-ori-1, PTC cell lines also exhibited a higher expression of CDH4. Intriguingly, differential expression levels were observed for CDH4 in various cellular compartments, including the cytomembrane, cytoplasm, and nucleus, with TPC-1 and BCPAP cell lines showing predominant cytoplasmic localization (Fig. 1G). According to the IHC score of CDH4, a remarkable association of CDH4 expression with lymph node metastasis (LNM) was discerned (*P* = 0.017; Table 1). However, no significant association was identified between CDH4 localization and clinicopathological features (*P* > 0.05; Additional file 1: Table S3). Hence, these results indicated that cytosolic CDH4 was upregulated and may exert an oncogenic role in PTC.

**Table 1:** Expression of CDH4 in PTC tissues according to clinicopathological information

Characteristic	Number	CDH4 expression		*P*-values^*^
		Low/Medium	High	
Age (years)				
≤ 55	36	20	16	
> 55	10	6	4	0.991
Sex				
Male	13	8	5	
Female	33	18	15	0.667
TNM stage				
I	42	23	19	
II	4	3	1	0.801
T stage				
T1a	19	11	8	
T1b	21	12	9	
T2/3	6	3	3	0.941
N stage				
Absent	23	17	6	
Present	23	9	14	**0.017**

^*^P values were calculated using the chi-square test. The significant results are in bold

### CDH4 promotes the metastasis, angiogenesis, and tumorigenesis of PTC

**Fig. 2:**
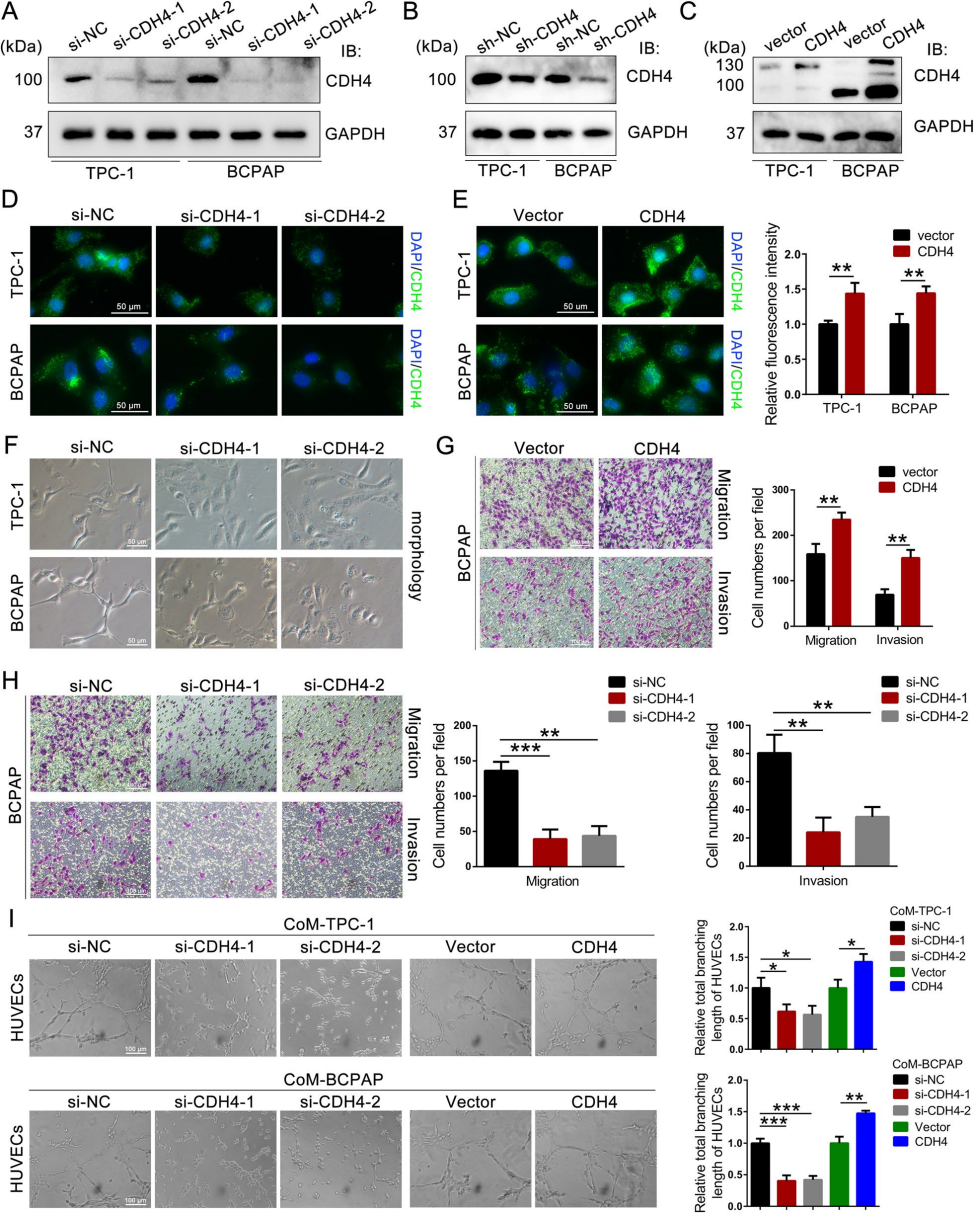
CDH4 promotes the migration, invasion, and angiogenesis of PTC cells. **A**, **B** Cellular CDH4 was effectively downregulated using CDH4-targeting siRNAs (**A**) or shRNA (**B**). **C** The expression of LPL was overexpressed in PTC cells by transfecting lentivirus expressing CDH4. **D** Representative images depicting the localization and intensity of CDH4 in PTC cells with suppressed CDH4 expression. **E** Immunofluorescence images (left) and quantification (right) of CDH4 in PTC cells with enhanced CDH4 expression. **F** Typical image showing the morphological changes of TPC-1 and BCPAP cells treated with CDH4-targeting siRNA. **G**, **H** Representative images and histogram analysis of cell migration and invasion following overexpression of CDH4 (**G**) or knockdown of CDH4 (**H**). **I** Representative images and quantification of the tube formation of HUVECs cocultured with conditional medium (CoM) derived from TPC-1 and BCPAP cells. *P < 0.05; **P < 0.01; ***P < 0.001

To investigate the role of cytosolic CDH4, TPC-1 and BCPAP cells were transfected with CDH4-targeting siRNA, or lentiviruses carrying control shRNA/empty vector, CDH4 shRNA, or CDH4 expressing vector (Fig. 2A–C). Immunofluorescence analysis also confirmed the efficacy of CDH4-targeting siRNA or expressing vector and further substantiated the presence of CDH4 expression across diverse cellular compartments (Fig. 2D, E; Additional file 1: Fig. S2A). Of note, noticeable morphological alterations were observed in TPC-1 and BCPAP cells following transduction with CDH4-targeting siRNA (Fig. 2F). In comparison to the wild-type cells, the cells treated with CDH4 siRNA exhibited a condensed appearance and adopted a cobblestone-like morphology. The transwell assay showed that the knockdown of CDH4 substantially impaired the migratory and invasive capabilities of PTC cells, whereas the overexpression of CDH4 exerted an opposite effect (Fig. 2G, H; Additional file 1: Fig. S2B, C). Co-culturing with conditioned media (CoM) from CDH4-silencing TPC-1 or BCPAP cells resulted in a reduction of tube formation in HUVECs compared to the control group (Fig. 2I). Conversely, co-culture with CoM derived from PTC cells overexpressing CDH4 led to an upregulation of tube formation (Fig. 2I).

**Fig. 3:**
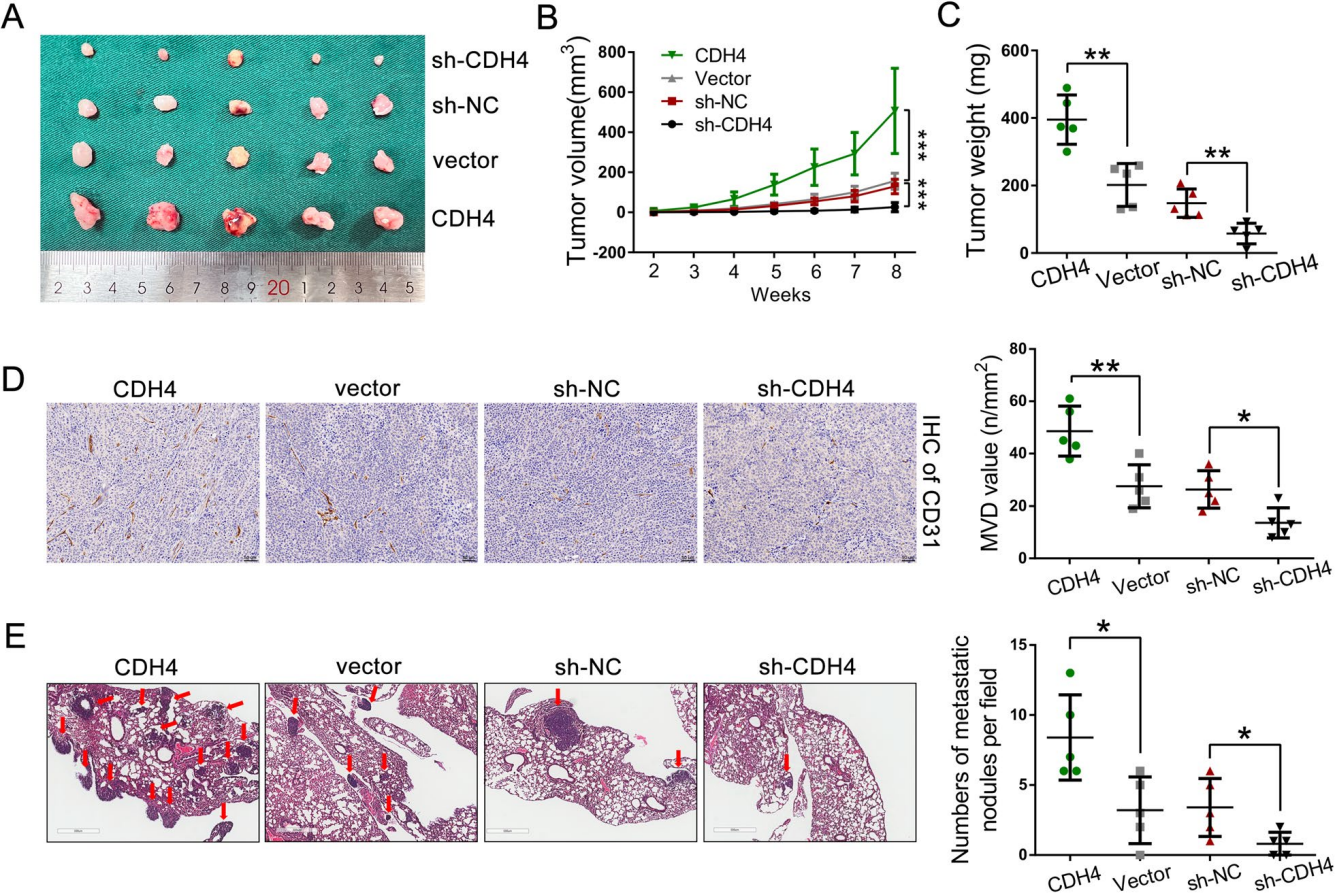
CDH4 promotes tumorigenesis and metastasis of PTC cells in vivo. **A** Representative image of the enucleated subcutaneous tumors from nude mice (n = 5 per group). **B**, **C** Growth curves (**B**) and final tumor weight (**C**) of each group were shown. **D** IHC analysis of CD31 in indicated enucleated subcutaneous tumors and the microvascular density (MVD) value was calculated for each group. **E** Images of HE staining to lung sections with metastatic sites and the number of lung metastatic nodules in each group were measured. *P < 0.05; **P < 0.01

To demonstrate the oncogenic role of CDH4 in vivo, we subcutaneously injected TPC-1 cells into the armpit of nude mice (Fig. 3A). Remarkably, the tumor formation rates were significantly decelerated in the CDH4-silencing groups compared to the control groups, and there was also a notable reduction in tumor weight observed in the CDH4-knockdown groups (Fig. 3B, C). Meanwhile, overexpression of CDH4 promoted tumor formation and enhanced the final tumor weight of subcutaneous tumors compared to the control group (Fig. 3B, C). Consistently, immunohistochemical analysis of subcutaneous tumors for angiogenesis marker platelet and endothelial cell adhesion molecule-1 (CD31) indicated decreased blood vessel formation in the CDH4-knockdown group and elevated levels in the CDH4-overexpressing group (Fig. 3D). Furthermore, we assessed the impact of CDH4 on PTC cell dissemination and metastatic colonization by directly inoculating TPC-1 cells into the tail vein of nude mice. Our findings revealed a decrease in lung metastatic burden in the CDH4-silencing group compared to the control, while an increase was observed in the CDH4-overexpressing group (Fig. 3E). Consequently, our results strongly suggest that CDH4 plays a crucial role in the angiogenesis, tumorigenesis, and metastasis of PTC.

### Cytosolic CDH4 interacts with β-catenin

**Fig. 4:**
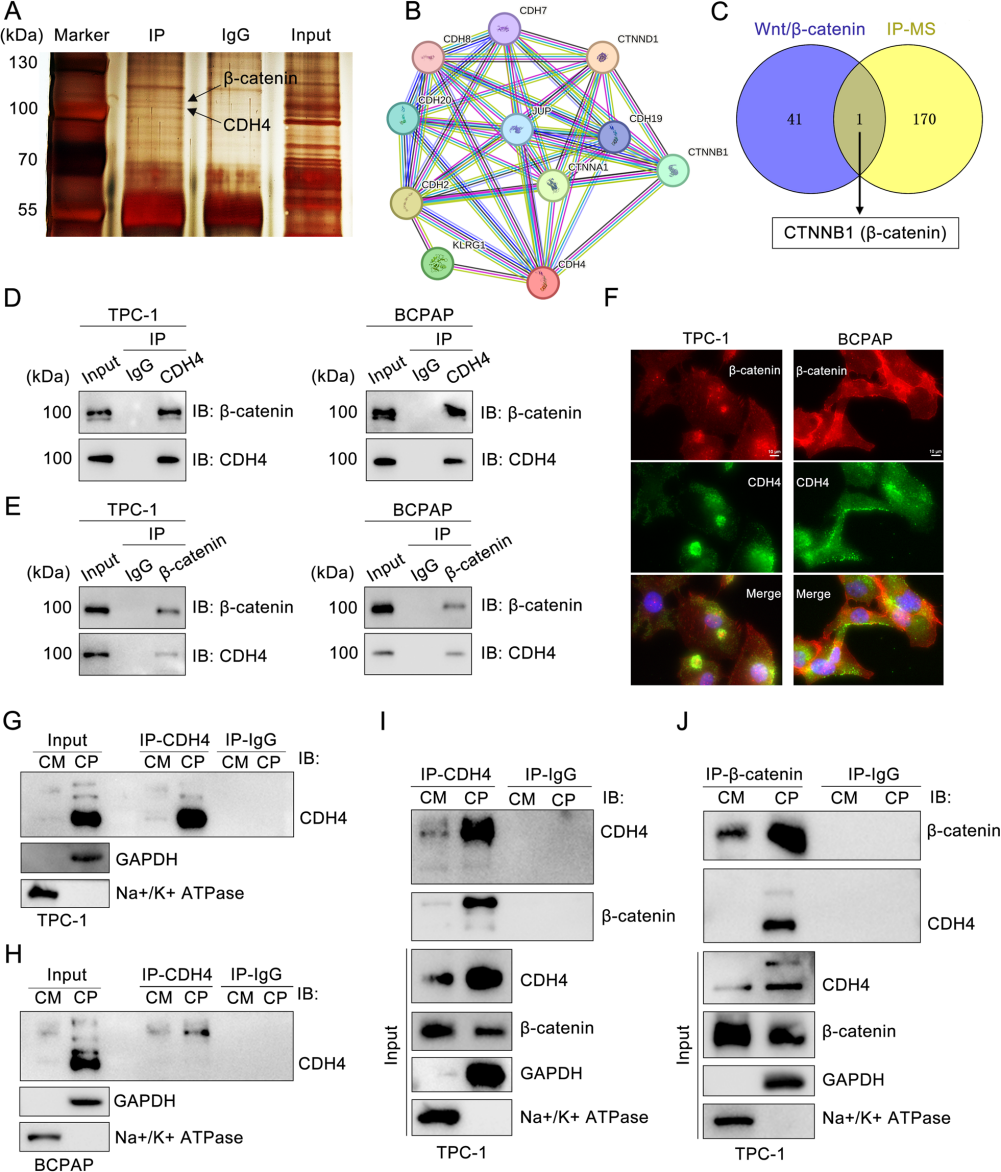
Cytosolic CDH4 interacts with β-catenin. **A** An IP assay was performed on TPC-1 cells with expression of His-CDH4, and the immunoprecipitates were separated using SDS-PAGE and stained with Silver. **B** STRING interaction network was conducted based on CDH4. **C** Venn diagram showing potential binding genes for CDH4.** D** IP analyses were performed with TPC-1 and BCPAP cells transiently transfected with His-CDH4 plasmids. **E** Extracts of PTC cells were immunoprecipitated with anti-β-catenin and control (IgG) antibodies. Co-immunoprecipitated proteins were detected via immunoblotting with their respective antibodies. **F** Immunofluorescence analysis showing colocalization of β-catenin (red) and CDH4 (green) in PTC cells. Nuclei were counterstained with DAPI (blue). **G**, **H** In TPC-1 (**G**) and BCPAP (**H**) cells, immunoprecipitation of CDH4 after cytosol-membrane fractionation was detected by western blot. The input was used for protein expression controls, GAPDH cytosol control, or Na + /K + ATPase membrane control. **I**, **J** Immunoprecipitation of CDH4 (**I**) or β-catenin (**J**) after cytosol-membrane fractionation in TPC-1 cells

To elucidate the functional mechanism of CDH4 in PTC, we introduced His-tagged CDH4 plasmids into TPC-1 cells. Subsequently, an immunoprecipitation assay was performed using the anti-His antibody, followed by silver staining and mass spectrometric analysis of the immunoprecipitates. As shown in Fig. 4A, there were two specific bands around the molecular weight of 100 kDa. According to the prediction of the STRING interaction network [25], CDH4 may interact with adhesion molecules and some other cadherins, such as junction plakoglobin, CDH2, and CTNBB1 (β-catenin) (Fig. 4B). Given the distinctive band observed and the cytoplasmic localization of CDH4, we postulated a potential interaction between CDH4 and β-catenin. Of note, we performed an intersection analysis between our mass spectrometric results and the Wnt signaling pathway, ultimately identifying β-catenin as a putative binding partner for CDH4 (Fig. 4C; Additional file 2: Table S4). WB analysis confirmed that CDH4 and β-catenin interact with each other (Fig. 4D, E; Additional file 1: Fig. S3A). Furthermore, immunofluorescence analysis showed that there was significant co-localization between CDH4 and β-catenin in PTC cells, especially in TPC-1 cells (Fig. 4F; Additional file 1: Fig. S3D). We also validated the subcellular distribution of CDH4 in the immunoprecipitation experiments (Fig. 4G, H). Consistently, we observed a successful pull-down of cytosolic β-catenin in the immunoprecipitates from cytosolic CDH4 (Fig. 4I; Additional file 1: Fig. S3B). Additionally, within the immunoprecipitates obtained from cytosolic β-catenin, we also detected the expression of CDH4 (Fig. 4J; Additional file 1: Fig. S3C). Therefore, these results showed that CDH4 interacts with β-catenin in the cytoplasm.

### Cytosolic CDH4 suppresses the ubiquitination/degradation of β-catenin

**Fig. 5:**
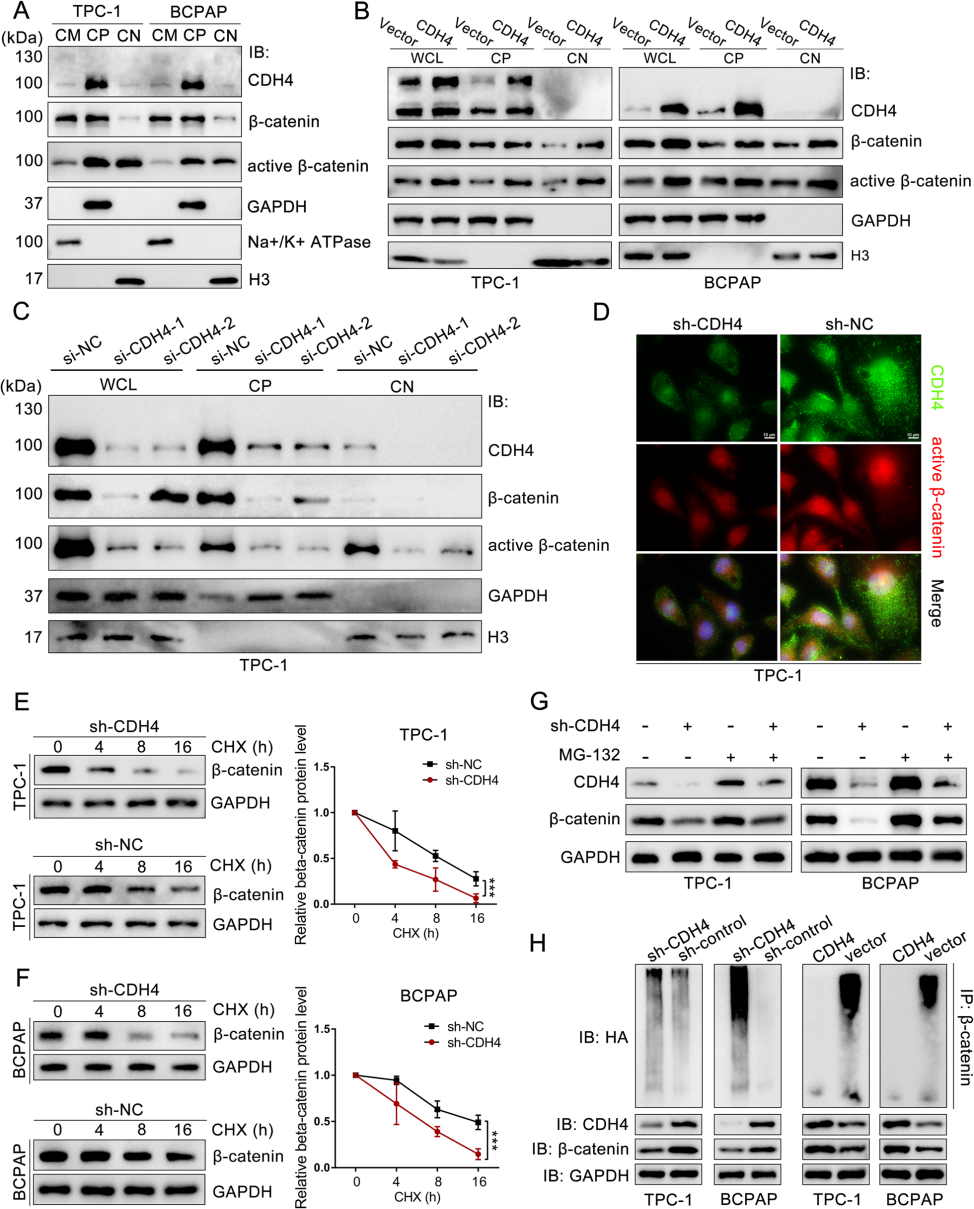
Cytosolic CDH4 suppresses the ubiquitination/degradation of β-catenin. **A** The expression of CDH4, β-catenin, and active β-catenin in the cytomembrane (CM), cytoplasm (CP), and cell nucleus (CN) was detected by WB. **B** Overexpression of CDH4 in PTC cells enhanced the expression of β-catenin and active β-catenin in the whole cell lysates, cytoplasm, and cell nucleus. **C** Knockdown of CDH4 inhibited the expression of β-catenin and active β-catenin. **D** Immunofluorescence analysis showing the decreased intensity of active β-catenin in TPC-1 cells transfected with CDH4 shRNA. **E**, **F** Half-life analysis of β-catenin abundance in CDH4 knockdown or control TPC-1 (**E**) and BCPAP (**F**) cells for the indicated periods in the presence of CHX (100 µM). The β-catenin level was normalized to GAPDH, and quantification of β-catenin stability is shown on the right. ***P < 0.001, two-way ANOVA test. **G** TPC-1 and BCPAP cells were transfected with CDH4 shRNA or a control vector for 48 h, followed by treatment with MG132 (20 μM) for an additional 6 h before WB analysis. **H** Immunoblotting analysis of the ubiquitination of endogenous β-catenin in PTC cells transfected with CDH4 shRNA, CDH4-overexpressing vector, or corresponding control vector

To investigate the effect of CDH4 on β-catenin, we first detect the expression of β-catenin or non-phospho β-catenin (active β-catenin) in the cytomembrane, cytoplasm, and cell nucleus [26]. As shown in Fig. 5A, β-catenin exhibited predominant expression in the cytomembrane and cytoplasm of PTC cells, whereas active β-catenin demonstrated prominent expression within the cytoplasmic and nuclear compartments. We subsequently observed that the overexpression of CDH4 markedly enhanced the expression levels of β-catenin and active β-catenin in both the cytoplasm and nucleus. Conversely, knockdown of CDH4 significantly attenuated their expression, particularly within the nucleus (Fig. 5B, C). We corroborate these results by immunofluorescence, which confirms the impaired nuclear localization of active β-catenin in CDH4 knockdown cells (Fig. 5D; Additional file 1: Fig. S3E). Consequently, our results demonstrate that cytosolic CDH4 can enhance β-catenin expression and facilitate its translocation into the nucleus.

Without the activation of the Wnt signal and was not bound to E-cadherin, β-catenin is phosphorylated in the cytoplasm, and phosphorylated β-catenin is recognized by β-TrCP1 and recruited to the E3 ubiquitin ligase complex, which was subsequently degraded by the 26S proteasome [20, 27]. Conversely, association with E-cadherin stabilizes β-catenin by impeding the interaction of β-catenin with components of the destruction complex. Hence, the upregulation of non-phospho β-catenin by CDH4 and the structural resemblance between CDH4 and E-cadherin have led us to infer that CDH4 may enhance β-catenin expression through inhibition of its ubiquitination. To validate this hypothesis, we initially assessed the half-life of β-catenin to investigate whether CDH4 regulates the stability of β-catenin protein. Following CDH4 knockdown, a significant decrease in the half-life of β-catenin was observed in PTC cells compared to that of the control groups (Fig. 5E, F). Subsequently, we discovered that CDH4-induced degradation of β-catenin was impeded by MG132, a proteasome inhibitor, in PTC cells (Fig. 5G), indicating that CDH4 exerts control over β-catenin protein stability via the proteasome. We then investigated the function of CDH4 on β-catenin protein ubiquitination in vivo by transfected CDH4 expressing vector or CDH4-targeting siRNA along with wild-type HA-Ub into PTC cells. The polyubiquitinated β-catenin was purified and then detected by IB with an anti-HA antibody. The results showed overexpression of CDH4 significantly attenuated the polyubiquitination of β-catenin (Fig. 5H). Conversely, the depletion of CDH4 in PTC cells stimulated the ubiquitination process of β-catenin (Fig. 5H). Therefore, our findings demonstrate that cytosolic CDH4 suppressed the ubiquitination of β-catenin, consequently leading to an enhancement in its nuclear expression.

### CDH4 interrupts the β-catenin–β-TrCP1 interaction

**Fig. 6:**
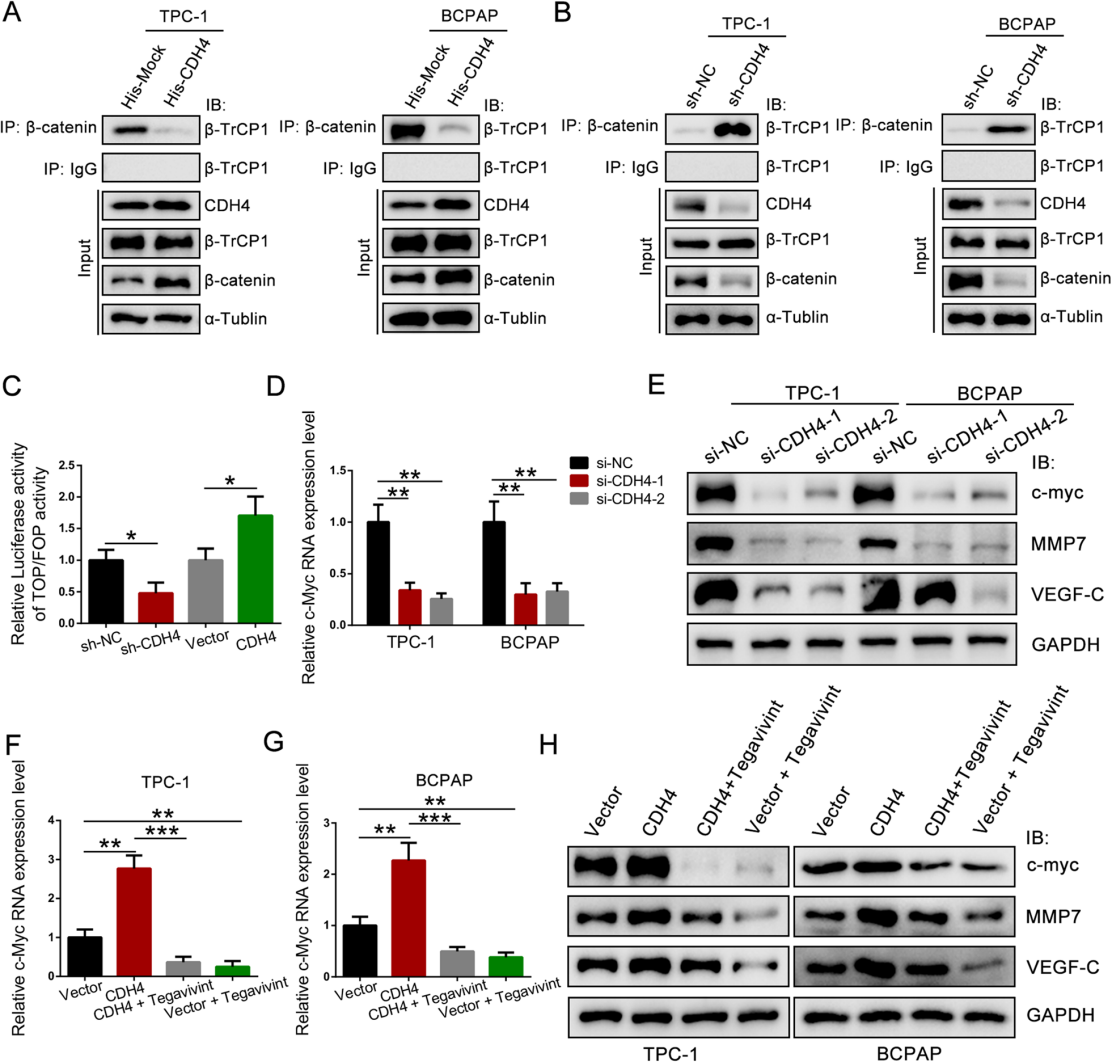
CDH4 interrupts the β-catenin–β-TrCP1 interaction and activates the transcription of downstream targets. **A** IP analyses were performed in TPC-1 and BCPAP cells transiently transfected with His-Mock (pcDNA3.1) or His-CDH4 plasmids, and the abundance of β-TrCP1 in the immunoprecipitates was detected. **B** IP analyses were performed in PTC cells transfected with CDH4 shRNA or control shRNA. **C** The effect of CDH4 on β-catenin signaling activity was evaluated by the TOP-flash/FOP-flash luciferase reporter assay. **D** The RNA level of c-Myc was detected in PTC cells transiently transfected with CDH4-targeting siRNA or control siRNA. **E** WB analysis of the protein level of c-Myc, MMP7, and VEGF-C in CDH4-silencing PTC cells. **F**, **G** TPC-1 (**F**) and BCPAP (**G**) cells overexpressing CDH4 were treated with Tegavivint (100 nmol/L, 24 h) as indicated, and qRT-PCR analysis of the RNA level of c-Myc was conducted. **H** Tegavivint reversed the effect of CDH4 on the expression of c-Myc, MMP7, and VEGF-C. *P < 0.05; **P < 0.01; ***P < 0.01

To assess whether CDH4 affects the association between β-catenin and the destruction complex, we performed IP with an anti-β-catenin antibody, followed by immune blotting (IB) with an anti-β-TrCP1 antibody. The results revealed that ectopic expression of CDH4 increased the β-catenin–β-TrCP1 interaction without altering the protein levels of β-TrCP1, while silencing of CDH4 decreased this interaction (Fig. 6A, B). Hence, cytosolic CDH4 disrupts the interaction between β-catenin and β-TrCP1, consequently impeding the ubiquitination process of β-catenin.

In the nucleus, β-catenin associates with transcription factors from the TCF/Lef family and orchestrates the transcriptional activation of Wnt/β-catenin target genes. Therefore, we then detected the expression levels of downstream targets of β-catenin, such as c-Myc, MMP7, and VEGF-C [28, 29]. Notably, the knockdown of CDH4 significantly attenuated the protein and RNA levels of c-Myc, MMP7, and VEGF-C (Fig. 6C–E; Additional file 1: Fig. S4A, B). Moreover, overexpression of CDH4 increased the expression of these genes; however, Tegavivint, an antagonist of β-catenin, could reverse this effect (Fig. 6F–H; Additional file 1: Fig. S4C, D). Thus, it showed CDH4 hinders the interaction between β-TrCP1 and β-catenin by directly binding to β-catenin, consequently impeding the ubiquitination of β-catenin. This inhibition ultimately facilitates the nuclear localization of β-catenin and triggers the transcription of downstream target genes.

### Tegavivint reverses the oncogenic role of CDH4 on PTC

**Fig. 7:**
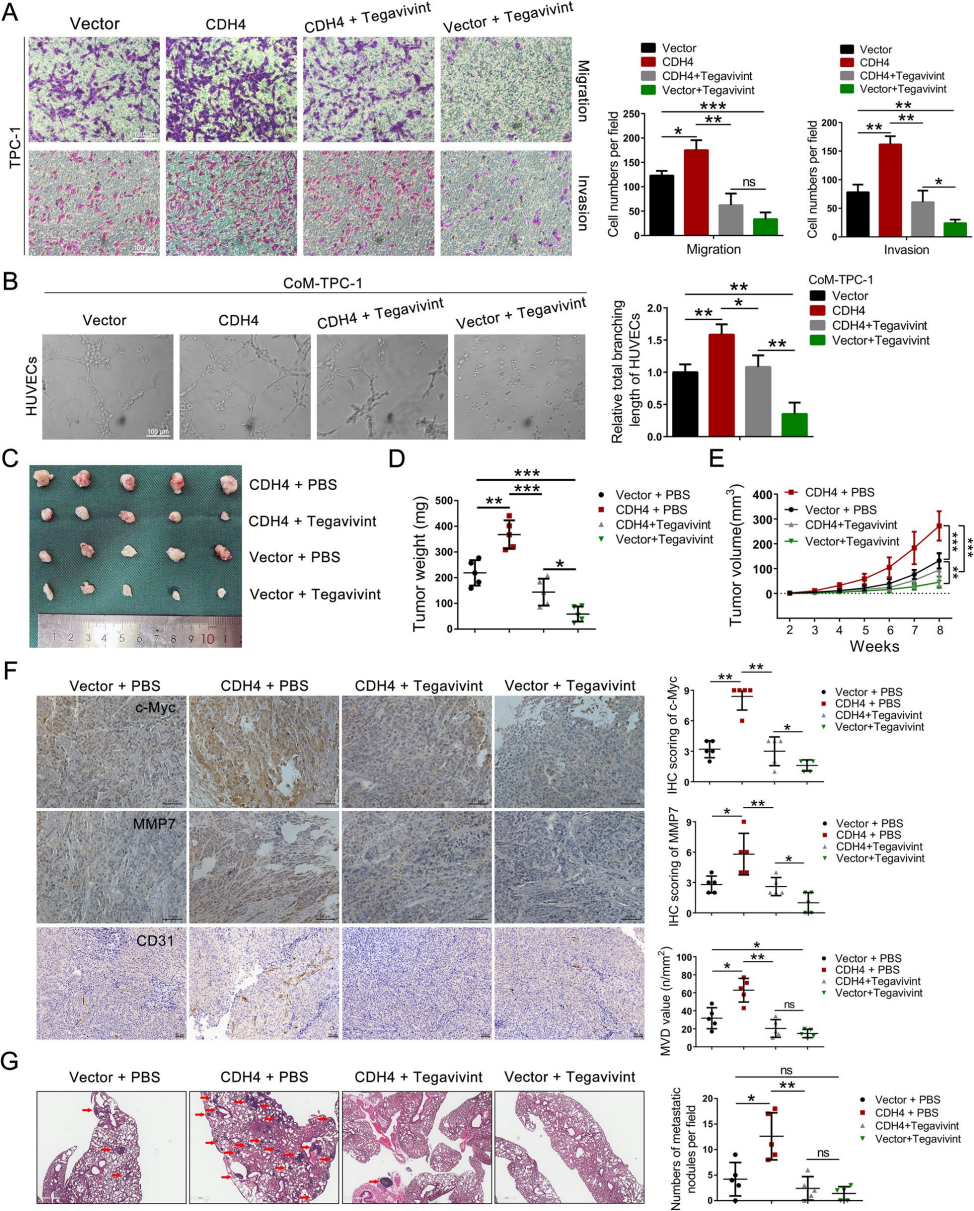
Tegavivint reverses the oncogenic role of CDH4 on PTC. **A** Representative image and quantification of the Transwell migration and invasion of TPC-1 cells, as indicated. **B** Representative images and quantification of the tube formation of HUVECs cocultured with conditional medium (CoM) derived from treated TPC-1 cells. **C**–**E** Representative images of the enucleated tumors (**C**) and presentation of the tumor volume (**E**) and final tumor weight (**D**) for each treated mice group (n = 5 per group). **F** Representative images (left) and histogram analysis (right) of the expression of c-Myc, MMP7, and CD31 in enucleated tumors. **G** HE staining images of lung sections with metastatic sites were obtained, and the quantification of lung metastatic nodules was performed for each experimental group. *P < 0.05; **P < 0.01; ***P < 0.01

To test whether CDH4 exerts its effects through β-catenin, we treated PTC cells with CDH4 expressing vector along with Tegavivint treatment (100 nmol/L, 24 h). Notably, Tegavivint effectively counteracted the pro-migratory and invasive effects of CDH4 on PTC cells (Fig. 7A; Additional file 1: Fig. S5A). Consistently, Tegavivint also significantly reversed the impact of CDH4 on tube formation in HUVECs (Fig. 7B; Additional file 1: Fig. S5B). These findings were further substantiated by our in vivo experiments. Specifically, we separately introduced CDH4-overexpressing TPC-1 cells or control cells into nude mice and administered intraperitoneal treatment with Tegavivint to the designated groups. Remarkably, treatment with Tegavivint significantly impeded tumor formation in CDH4-overexpressing TPC-1 cells (Fig. 7C), and it also significantly reduced the volume and weight of tumors compared to both control and CDH4-overexpressing groups (Fig. 7D, E). Importantly, immunohistochemical analysis of c-Myc, MMP7, and CD31 in collected subcutaneous tumors revealed that CDH4 overexpression led to an upregulation of these proteins; however, this effect was reversed by Tegavivint (Fig. 7F). Furthermore, the lung metastatic model also showed Tegavivint could impaired CDH4-induced lung metastasis (Fig. 7G). Meanwhile, immunohistochemical analysis of β-catenin in collected subcutaneous tumors also confirmed the positive regulatory role of CDH4 on β-catenin (Additional file 1: Fig. S5C, D). Collectively, these findings strongly support the notion that CDH4 promotes tumorigenesis and metastasis of PTC by enhancing the stability of β-catenin, which can be effectively inhibited by Tegavivint.

## Discussion

Distant metastasis is responsible for the majority of deaths in patients diagnosed with differentiated thyroid cancer (DTC), which includes PTC and follicular thyroid carcinoma [30]. Distant metastasis occurs in 4–23% of patients with DTC, with the lung being the most common site for distant spread [31]. The 10-year survival rate following a diagnosis of metastatic DTC ranges from 25 to 70%, and patients with metastatic DTC exhibit diverse clinical outcomes, ranging from rapid progression and mortality to complete remission, indicating significant heterogeneity among these individuals [31, 32]. Hence, it is imperative to investigate and scrutinize the molecular mechanism underlying PTC metastasis. In this study, we discovered that CDH4 plays a pivotal role in promoting invasion, metastasis, and angiogenesis in PTC. Furthermore, the overexpression of CDH4 in tumor tissues is significantly associated with lymph node metastasis in PTC patients. These findings strongly suggest that CDH4 may serve as a crucial driver for PTC metastasis, thereby offering valuable insights for future diagnosis and treatment strategies targeting PTC metastasis.

In recent years, several studies have unveiled the oncogenic or suppressive role of CDH4 in diverse cancer types. For instance, the knockout of CDH4 significantly diminished the malignant properties of bladder cancer cells [33]. In osteosarcoma, overexpression of CDH4 triggers the activation of c-Jun through the JNK pathway and fosters tumor xenograft growth and lung colonization [16]. Conversely, aberrant methylation of the CDH4 gene promoter suggests that CDH4 may also function as a tumor suppressor gene in human nasopharyngeal, colorectal, and gastric cancer [14, 34]. In this study, we found that cytosolic CDH4 plays a pivotal role in promoting PTC metastasis and progression. Our findings offer potential avenues for future investigations into CDH4, including the exploration of the impact of cytoplasmic versus membranal CDH4 on tumor development, investigation into possible interactions between CDH4 and other cadherins, and examination of how the tumor microenvironment influences CDH4 expression and localization.

EMT is one of the hallmarks of tumor metastasis, and cadherins play a pivotal role in this process [35]. Cancer cells undergoing EMT display both morphological and molecular alterations [36], which is evidenced by the downregulation of epithelial markers, such as E-cadherin and ZO-1, and upregulation of mesenchymal markers, including N-cadherin, vimentin, fibroblast-specific protein 1, and fibronectin [35]. Additionally, various signaling pathways are implicated in the regulation of EMT, such as the Wnt/β-catenin and Notch pathways [35, 37]. In this study, the knockdown of CDH4 in PTC cells induced a mesenchymal-like morphology while cytosolic CDH4 directly activated β-catenin signaling. These findings suggest that CDH4 may play a role in the EMT process associated with PTC metastasis. In addition, cadherin switching contributes to tumor metastasis, characterized by the transition from E-cadherin to N-cadherin or a reduction in E-cadherin levels accompanied by an upregulation of P- and R-cadherin expression within tumor tissues [33, 37, 38]. However, no investigations have been conducted thus far regarding the potential co-expression switch between CDH4 and other cadherin proteins in PTC. Of note, in this study, we also observed a downregulation of E-cadherin expression and an up-regulation of CDH4 expression in PTC tissues. However, further analysis is required to establish a potential correlation between these two phenomena.

Although the role of CDH4 in tumors remains elusive, several studies have indicated that CDH4 can promote tumorigenesis and metastasis in osteosarcoma and bladder cancer [16, 33]. Martins‑Lima et al. found that the expression of membranal CDH4 was significantly increased in bladder urothelial carcinoma tissues [33]. However, our findings demonstrate an overexpression of cytosolic CDH4 rather than membrane-localized CDH4 within PTC tissues, highlighting the versatile role and context-dependent phenotype of CDH4 in human cancers. In general, the steady-state levels of cadherin at the plasma membrane are determined by the rates of endocytosis and degradation, which lead to a decrease in surface levels [39]. Conversely, the synthesis of new proteins and recycling contribute to an increase in cadherin availability at the plasma membrane [39]. For instance, E-cadherin primarily undergoes internalization or degradation through clathrin-mediated endocytosis, a process that is negatively regulated by p120-catenin [40]. Thus, elucidating the mechanism by which CDH4 transitions from membrane to cytoplasmic localization in PTC is an important topic. Notably, we observed that CDH4 exhibits two bands at molecular weights of 100 kDa and 130 kDa under specific conditions (Fig. 2C). Previous studies have demonstrated the susceptibility of cadherins to proteolytic cleavage, resulting in fragments of varying sizes [41–43]. Su et al. further reported the calpain-mediated cleavage of VE-cadherin upon entry into clathrin-enriched domains, generating a ∼ 95-kDa fragment [44]. Importantly, this process is dependent on endocytosis and subsequently affects the trafficking dynamics and degradation of VE-cadherin [44]. Therefore, it is plausible that a cleaved form (100 kDa) of CDH4 may represent its active state within the cytosol of PTC cells. However, to ascertain such an occurrence, it is imperative to determine the molecular weight of full-length CDH4 first and subsequently investigate any evidence of proteolysis affecting CDH4 protein integrity.

The interaction between cadherins and β-catenin has been extensively documented, whereas the specific investigation of the interaction between CDH4 and β-catenin remains lacking [45]. Our study indicated that cytosolic CDH4 disrupts the interaction between β-catenin and β-TrCP1, consequently impeding the ubiquitination process of β-catenin. In melanoma, Delgado-Bellido et al. found that nuclear VE-cadherin is associated with β-catenin, resulting in decreased β-catenin degradation while enhancing TCF4-dependent gene transcription [46]. In colorectal cancer, overexpression of receptor-interacting protein kinase 1 (RIP1) can destroy the β-catenin–β-TrCP complex and therefore promotes EMT [47]. Hence, the interaction mechanisms between cadherins and β-catenin are diverse [48]. Our study presents a novel perspective on the regulation of β-catenin by cadherins.

## Conclusions

Taken together, our data suggest that cytosolic CDH4 inhibited β-catenin’s proteasomal degradation and increased its transcriptional activation of c-Myc, MMP7, and VEGF-C, thereby modulating PTC angiogenesis and metastasis. Therefore, if the cytoplasmic localization of CDH4 is perturbed, it could potentially impede the activation of β-catenin signaling, thereby exerting inhibitory effects on tumor metastasis.

### Supplementary Information

**Additional file 1: Figure S1.** IHC staining of CDH4 in 29 paired papillary thyroid cancer tissues and adjacent normal tissues. A IHC staining of CDH4 in a tissue microarray containing 29 paired papillary thyroid cancer tissues and adjacent normal tissues. **Figure S2.** CDH4 promoted the cell migration and invasion of TPC-1 cell line. **A** Quantification of the immunofluorescence intensity of CDH4 in TPC-1 and BCPAP cells transfected with CDH4-tagerting siRNA or control siRNA. **B**, **C** Representative images and histogram analysis of TPC-1 cell migration and invasion following overexpression of CDH4 (**B**) or knockdown of CDH4 (**C**). *P < 0.05; **P < 0.01. **Figure S3.** Cytosolic CDH4 interacted with β-catenin in BCPAP cells. **A** Mass spectrometry image of β-catenin. **B**, **C** Immunoprecipitation of CDH4 (**B**) or β-catenin (**C**) after cytosol-membrane fractionation in BCPAP cells. **D** Quantification of colocalization between CDH4 and β-catenin in PTC cells. **E** Quantification of the immunofluorescence intensity of CDH4 and active β-catenin in TPC-1 transfected with CDH4-tagerting shRNA or control shRNA. **P < 0.01. **Figure S4.** CDH4 regulated β-catenin-dependent transcriptional activation of MMP7 and VEGF-C. **A**, **B** The RNA level of MMP7 (**A**) and VEGF-C (**B**) was detected in PTC cells transiently transfected with CDH4-targeting siRNA or control siRNA. **C**, **D** PTC cells overexpressing CDH4 were treated with Tegavivint (100 nmol/L, 24 h) as indicated, and qRT-PCR analysis of the RNA level of MMP7 (**C**) and VEGF-C (**D**) was conducted. *P < 0.05; **P < 0.01; ***P < 0.01. **Figure S5.** Tegavivint reversed the oncogenic role of CDH4 on BCPAP cells. **A** Representative images and quantification of the transwell migration and invasion of BCPAP cells, as indicated. **B** Representative images and quantification of the tube formation of HUVECs cocultured with conditional medium derived from treated BCPAP cells. **C**, **D** IHC staining of β-catenin in the transplanted tumor tissues. *P < 0.05; **P < 0.01; ***P < 0.01. **Table S1.** siRNA and shRNA used in this study. **Table S2.** Antibodies used in this study. **Table S3.** Cellular location of CDH4 in PTC tissues according to clinicopathological information.

**Additional file 2: Table S4.** Identified proteins in the mass spectrometric analysis.

## Data Availability

All data are present in the manuscript and the Supplementary Materials. Additional data related to this paper may be requested from the corresponding author.

## Abbreviations

PTC: Papillary thyroid cancer
DTC: Differentiated thyroid cancer
CDH4: Cadherin 4
c-Myc: MYC proto-oncogene, bHLH transcription factor
MMP7: Matrix metallopeptidase 7
VEGF-C: Vascular endothelial growth factor C
GAPDH: Glyceraldehyde-3-phosphate dehydrogenase
CD31: Platelet and endothelial cell adhesion molecule-1
CTNBB1: β-Catenin
EMT: Epithelial-to-mesenchymal transition
HUVECs: Human umbilical vein endothelial cells
qRT-PCR: Quantitative real-time PCR
IF: Immunofluorescence
IHC: Immunohistochemistry
WCL: Whole cell lysates
CM: Cytomembrane
CP: Cytoplasm
CN: Cell nucleus
IP: Immunoprecipitation
CoM: Conditioned media
IB: Immune blotting
β-TrCP: β-Transducin repeat-containing protein
