# Supplementary material for: Cytosolic Cadherin 4 promotes angiogenesis and metastasis in papillary thyroid cancer by suppressing the ubiquitination/degradation of β-catenin
Source: J Transl Med. 2024 Feb 24;22:201. doi: 10.1186/s12967-024-05012-1 (PMC10894493; doi:10.1186/s12967-024-05012-1)
Supplement: Supplementary file 1 — Additional file 1: Figure S1. IHC staining of CDH4 in 29 paired papillary thyroid cancer tissues and adjacent normal tissues. A IHC staining of CDH4 in a tissue microarray containing 29 paired papillary thyroid cancer tissues and adjacent normal tissues. Figure S2. CDH4 promoted the cell migration and invasion of TPC-1 cell line. A Quantification of the immunofluorescence intensity of CDH4 in TPC-1 and BCPAP cells transfected with CDH4-tagerting siRNA or control siRNA. B, C Representative images and histogram analysis of TPC-1 cell migration and invasion following overexpression of CDH4 (B) or knockdown of CDH4 (C). *P < 0.05; **P < 0.01. Figure S3. Cytosolic CDH4 interacted with β-catenin in BCPAP cells. A Mass spectrometry image of β-catenin. B, C Immunoprecipitation of CDH4 (B) or β-catenin (C) after cytosol-membrane fractionation in BCPAP cells. D Quantification of colocalization between CDH4 and β-catenin in PTC cells. E Quantification of the immunofluorescence intensity of CDH4 and active β-catenin in TPC-1 transfected with CDH4-tagerting shRNA or control shRNA. **P < 0.01. Figure S4. CDH4 regulated β-catenin-dependent transcriptional activation of MMP7 and VEGF-C. A, B The RNA level of MMP7 (A) and VEGF-C (B) was detected in PTC cells transiently transfected with CDH4-targeting siRNA or control siRNA. C, D PTC cells overexpressing CDH4 were treated with Tegavivint (100 nmol/L, 24 h) as indicated, and qRT-PCR analysis of the RNA level of MMP7 (C) and VEGF-C (D) was conducted. *P < 0.05; **P < 0.01; ***P < 0.01. Figure S5. Tegavivint reversed the oncogenic role of CDH4 on BCPAP cells. A Representative images and quantification of the transwell migration and invasion of BCPAP cells, as indicated. B Representative images and quantification of the tube formation of HUVECs cocultured with conditional medium derived from treated BCPAP cells. C, D IHC staining of β-catenin in the transplanted tumor tissues. *P < 0.05; **P < 0.01; ***P < 0.01. Table S1. siRNA and shRN [file 12967_2024_5012_MOESM1_ESM.pdf]

Figure S1-5 and Table S1-3

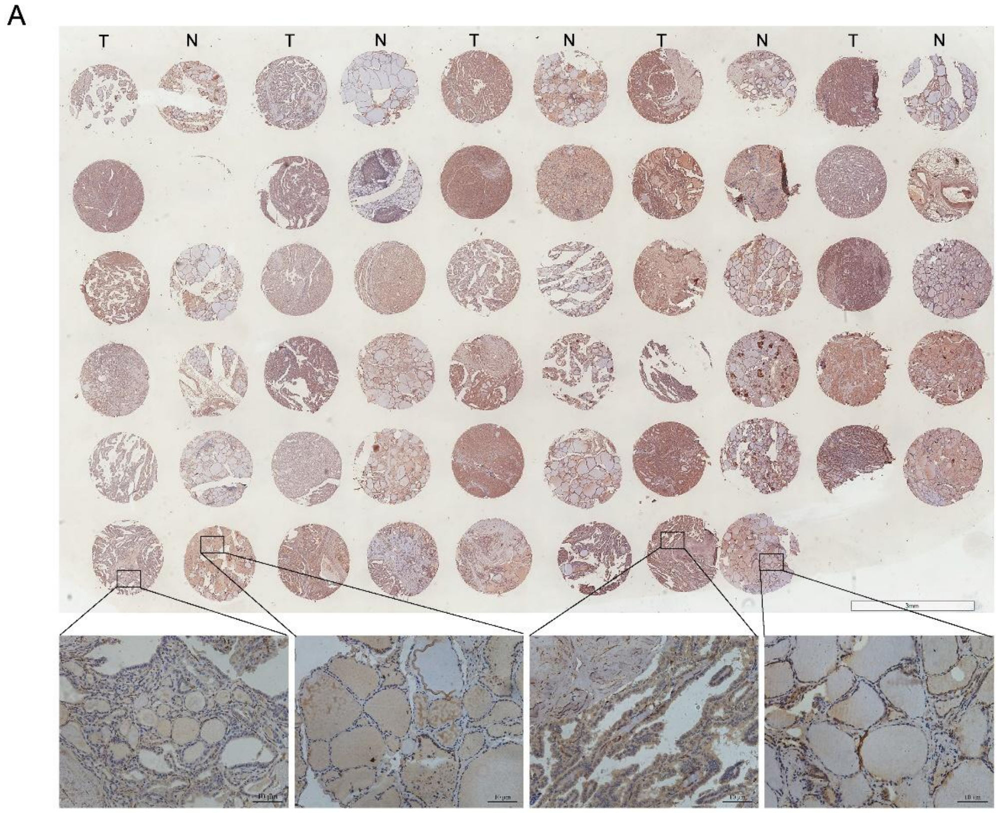

Figure S1 IHC staining of CDH4 in 29 paired papillary thyroid cancer tissues and adjacent normal tissues. **A** IHC staining of CDH4 in a tissue microarray containing 29 paired papillary thyroid cancer tissues and adjacent normal tissues.

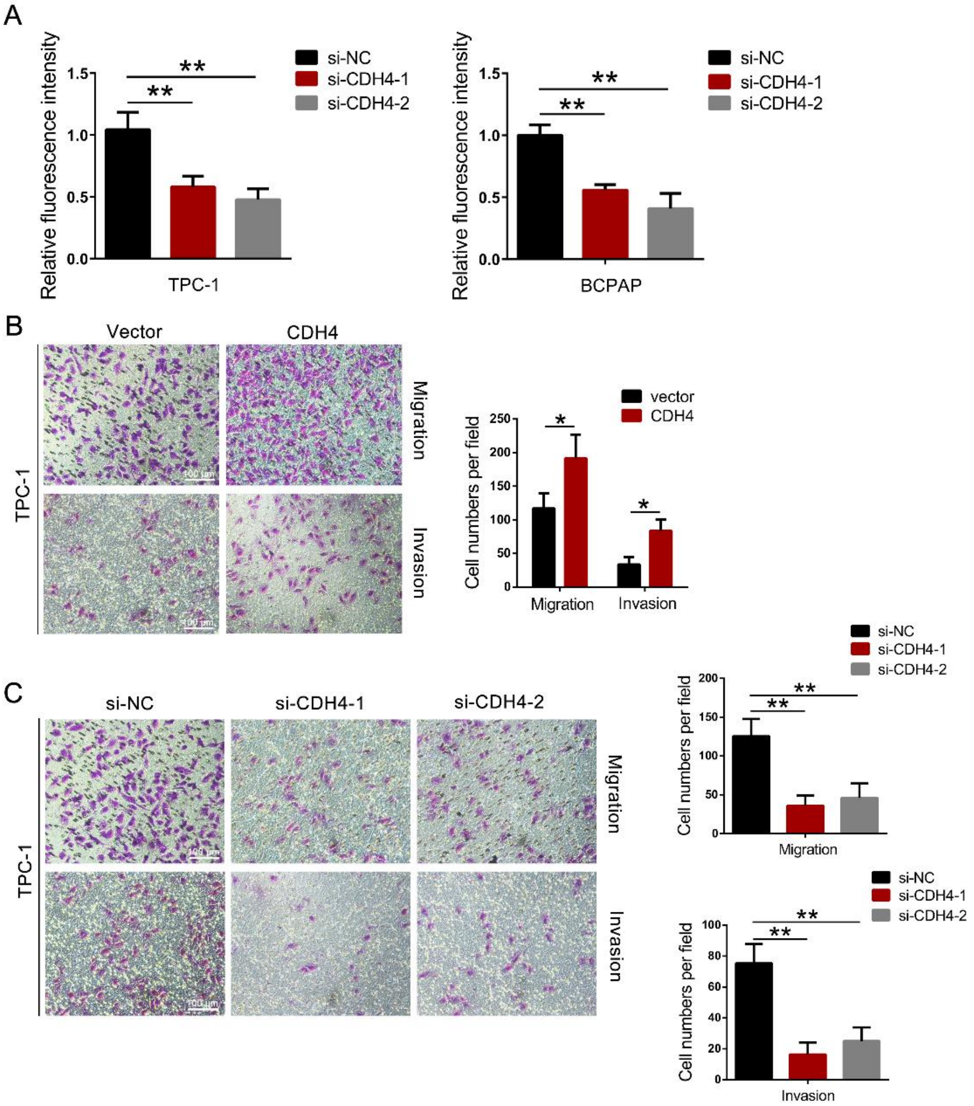

Figure S2 CDH4 promoted the cell migration and invasion of TPC-1 cell line. **A** Quantification of the immunofluorescence intensity of CDH4 in TPC-1 and BCPAP cells transfected with CDH4-tagerting siRNA or control siRNA. **B-C** Representative images and histogram analysis of TPC-1 cell migration and invasion following overexpression of CDH4 (**B**) or knockdown of CDH4 (**C**). *, P <0.05; **, P < 0.01.

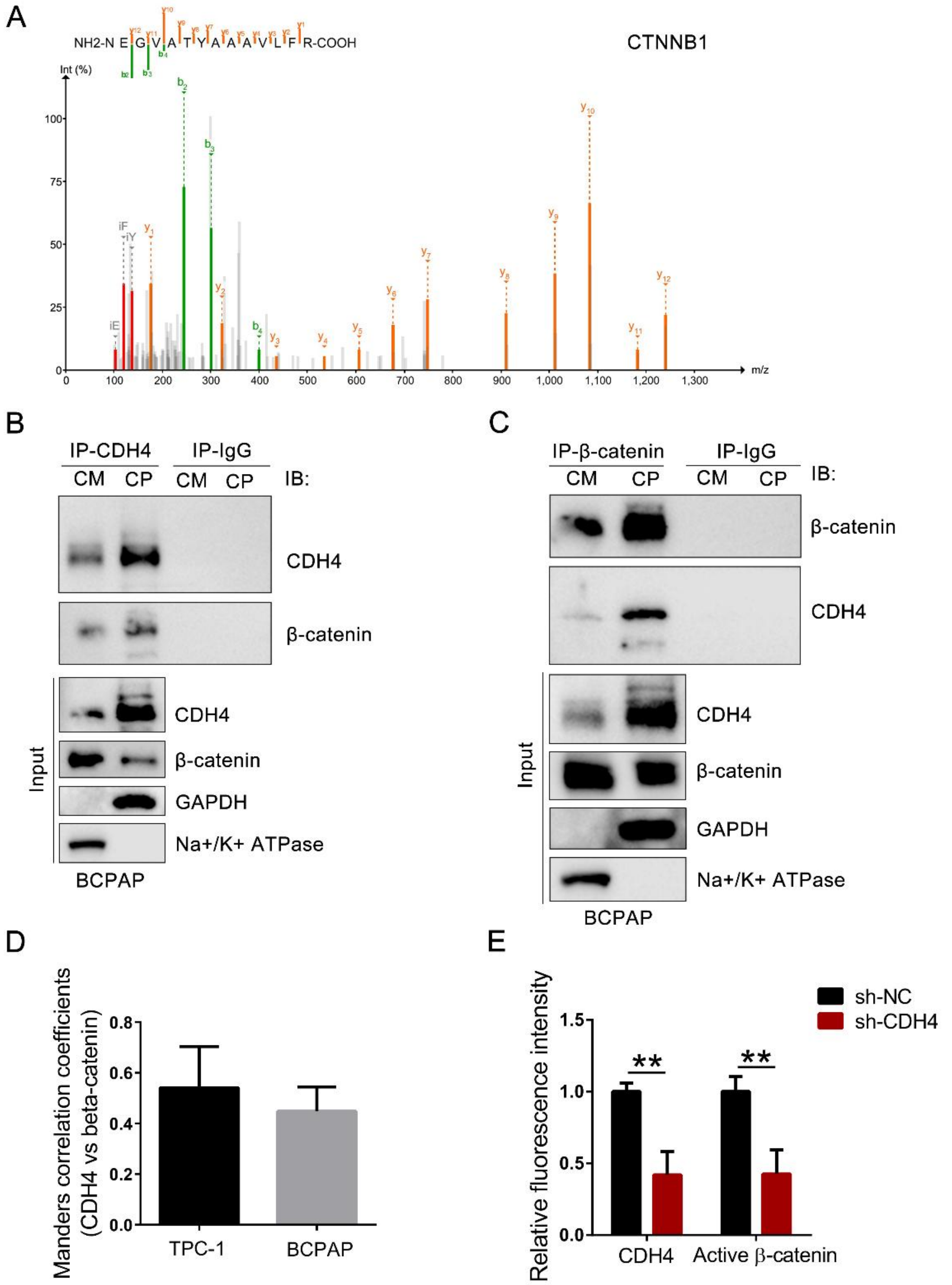

Figure S3 Cytosolic CDH4 interacted with β-catenin in BCPAP cells. **A** Mass spectrometry image of β-catenin. **B-C** Immunoprecipitation of CDH4 (**B**) or β-catenin (**C**) after cytosol-membrane fractionation in BCPAP cells. **D** Quantification of colocalization between CDH4 and β-catenin in PTC cells. **E** Quantification of the immunofluorescence intensity of CDH4 and active β-catenin in TPC-1 transfected with CDH4-tagerting shRNA or control

shRNA. **, $P < 0.01$.

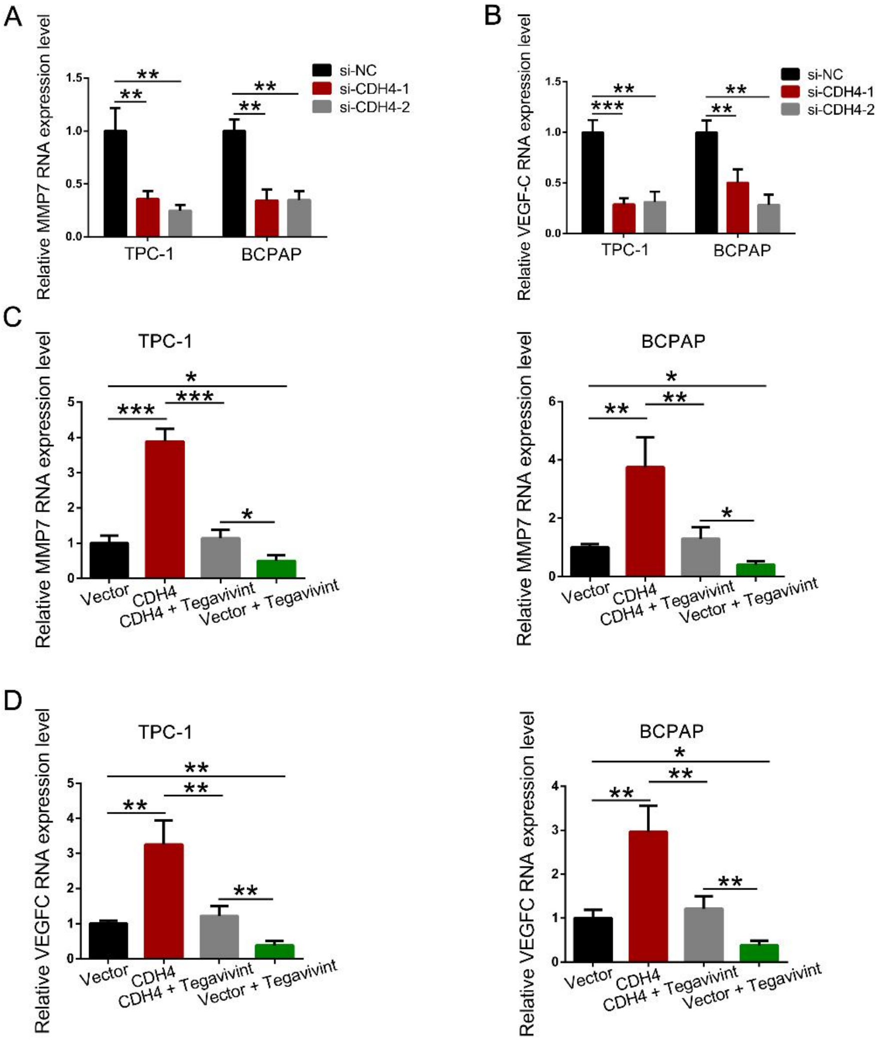

Figure S4 CDH4 regulated β-catenin-dependent transcriptional activation of MMP7 and VEGF-C. **A-B** The RNA level of MMP7 (**A**) and VEGF-C (**B**) was detected in PTC cells transiently transfected with CDH4-targeting siRNA or control siRNA. **C-D** PTC cells overexpressing CDH4 were treated with Tegavivint (100 nmol/L, 24h) as indicated, and qRT-PCR analysis of the RNA level of MMP7 (**C**) and VEGF-C (**D**) was conducted. *, P <0.05; **, $P < 0.01$; ***, $P < 0.01$.

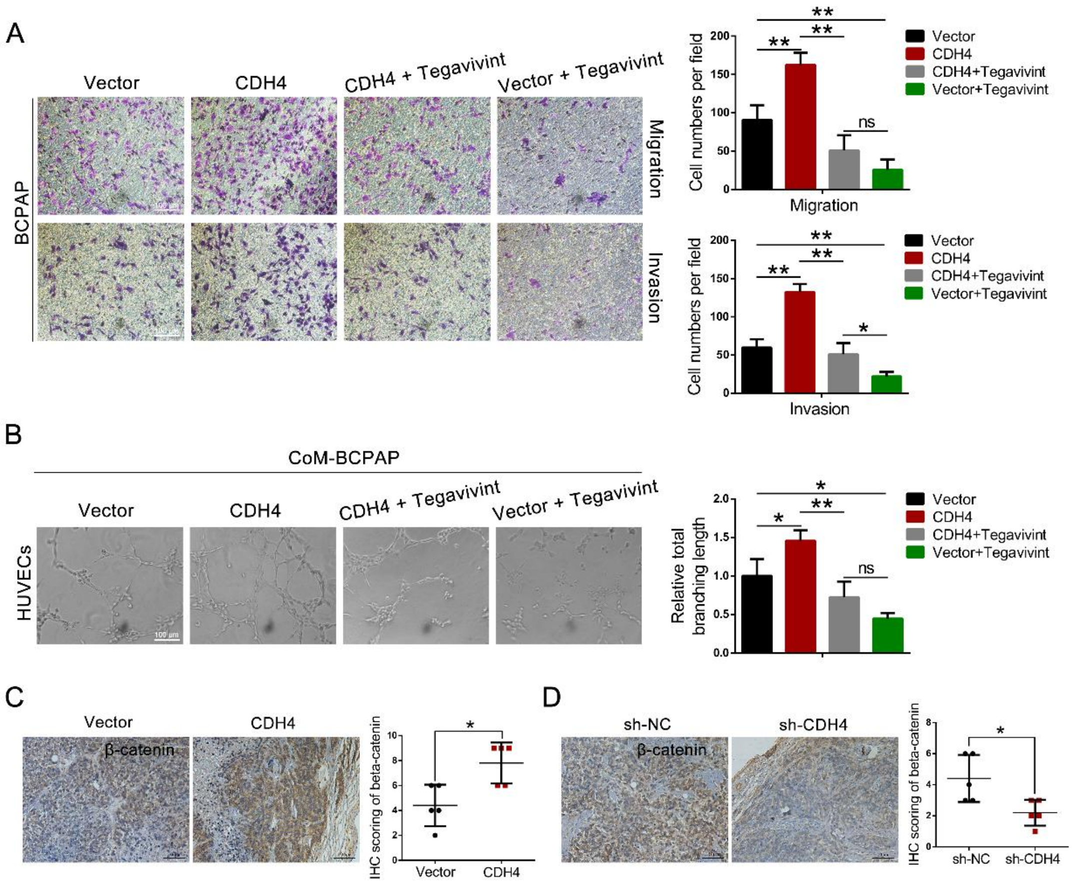

Figure S5 Tegavivint reversed the oncogenic role of CDH4 on BCPAP cells. **A** Representative images and quantification of the transwell migration and invasion of BCPAP cells, as indicated. **B** Representative images and quantification of the tube formation of HUVECs cocultured with conditional medium derived from treated BCPAP cells. **C-D** IHC staining of β-catenin in the transplanted tumor tissues. *, P <0.05; **, P < 0.01; ***, P < 0.01.

Table S1 siRNA and shRNA used in this study.

	5' to 3'
si-CDH4-1	CCACGUUUUCAGCUGUGGATT
si-CDH4-2	CAAGGCUGGGUUCUCUGAAGTT
si-NC	UUCUCCGAACGUGUCACGUTT
sh-CDH4	GGACACAAUAUGAGACCAACA
sh-NC	UUCUCCGAACGUGUCACGU

Table S2 Antibodies used in this study.

Name	Company	Host	Catalog Number	dilution	Applications
CDH4	Bioworld	Mouse	MB62763	1:1000	WB
				1:50	IF
				1: 400	IHC
E-cadherin	Proteintech	Mouse	60335-1-Ig	1:4000	WB
				1:10000	IHC
GAPDH	Proteintech	Mouse	60004-1-Ig	1:50000	WB
α-Tublin	Proteintech	Mouse	66031-1-Ig	1:10000	WB
Histone H3	Beyotime	Mouse	AF0009	1:1000	WB
Na+/K+ ATPase	abcam	Rabbit	ab76020	1:50000	WB
CD31	Beyotime	Rabbit	AG2849	1:400	IHC
β-catenin	Proteintech	Rabbit	51067-2-AP	1:4000	WB
				1:200	IF
Active β-catenin	abcam	Rabbit	ab246504	1:1000	WB
				1:100	IF
β-TrCP	Proteintech	Rabbit	28393-1-AP	1:1000	WB
VEGF-C	Beyotime	Rabbit	AF8325	1:1000	WB
c-Myc	Proteintech	Mouse	67447-1-Ig	1:2000	WB
				1:500	IHC
MMP7	Proteintech	Rabbit	10374-2-AP	1:1000	WB
				1:100	IHC

Table S3 Cellular location of CDH4 in PTC tissues according to clinicopathological information.

		CDH4 Location		
Characteristic	Number	CM	CP	*P*-values*
Age (years)				
≤ 55	36	10	26	
> 55	10	3	7	0.99
Sex				
Male	13	5	8	
Female	33	8	25	0.335
TNM stage				
I	42	13	29	
II	4	0	4	0.464
T stage				
T1a	19	6	13	
T1b	21	5	16	
T2/3	6	2	4	0.824
N stage				
Absent	23	6	17	
Present	23	7	16	0.743

* *P* values were calculated using the chi-square test.
